# Association of the immature platelet fraction with the diagnosis and severity of sepsis: an observational study

**DOI:** 10.1186/cc12990

**Published:** 2013-11-05

**Authors:** Melina V Rodrigues, Bruna D Andreguetto, Thiago M Santos, Maria de Fátima P Gilberti, Joyce M Annichino-Bizzacchi, Desanka Dragosavac, Marco A Carvalho-Filho, Erich V De Paula

**Affiliations:** 1 Faculty of Medical Sciences, University of Campinas, SP, Brazil

## Background

An ideal sepsis biomarker should be able to segregate infected patients from other causes of SIRS, and also to allow some kind of risk stratification. Furthermore, it should be capable of identifying subgroups of patients with specific sepsis complications, enabling target-specific preventive and therapeutic measures. Finally, access to this biomarker should not depend on complex and high-cost equipments and reagents, allowing access to more patients. New hematologic automated analyzers used for evaluation of the complete blood count provide a series of advanced analytical parameters that permit more detailed evaluations of circulating blood cells. Parameters such as the immature reticulocyte fraction (IRF) and immature platelet fraction (IPF) identify early signs of hematopoietic recovery, and have been studied in several inflammatory conditions. Recently, a study performed in critically ill patients suggested that the IPF could be a more accurate biomarker of sepsis development than C-reactive protein (CRP) and procalcitonin. The aim of this study was to evaluate whether IPF and IRF levels presented any association with clinical and laboratory parameters of sepsis severity.

## Materials and methods

During 30 days the IPF and IRF were obtained using an automated hematologic analyzer (Sysmex XE5000) within 24 hours from admission for consecutive patients with sepsis.

## Results

In total, 23 patients with sepsis were enrolled in the study, of which 12 (52%) presented severe sepsis or septic shock. The median APACHE II and SOFA scores at admission were 15 (6 to 37) and 6 (1 to 17). Median IPF and IRF levels at admission were 4% (1.1 to 11.0%) and 14% (1.6 to 47.1%) respectively, and were significantly higher than in a population of healthy individuals (IPF = 2.1% and IRF = 2.9%; both *P *< 0.001). As opposed to the CRP, both IPF and IRF were significantly correlated with the SOFA at admission (*R*s = 0.52 and 0.45; *P *= 0.01 and 0.02 respectively). However, when patients were stratified by the median SOFA score at admission, only the IPF was significantly higher in patients with SOFA ≥6 (IPF = 6.2% vs. 2.9%; *P *= 0.01). Similar results were observed when patients were stratified by the presence of severe sepsis. The IPF presented a significant correlation with the platelet count (*R*s = -0.71; *P *< 0.001), but with not with PT, aPTT and D-dimer.

## Conclusions

In patients with sepsis, both IPF and IRF were higher than in healthy individuals, and the IPF was associated with increased sepsis severity. Larger studies are warranted to define and validate the precise role of the IPF as a sepsis biomarker.

